# Gray matter macrostructure and brain aging in unhealthy alcohol users

**DOI:** 10.1192/j.eurpsy.2025.1665

**Published:** 2025-08-26

**Authors:** M. Hermesdorf, K. Berger

**Affiliations:** 1 Institute of Epidemiology and Social Medicine, University of Münster, Münster, Germany

## Abstract

**Introduction:**

In view of recent global trends in alcohol use, it becomes increasingly relevant to characterize health outcomes related to unhealthy alcohol use. Previous studies found that self-reported alcohol use was related to poor brain health. However, these studies remain inconclusive since they limited their analyses to very narrow demographic strata, considered only a subset of cortical regions, or didn’t validate self-reported alcohol use with biomarkers such as gamma-glutamyltransferase (gamma-GT).

**Objectives:**

This study aimed to comprehensively examine several aspects of brain health (cortical thickness, gray matter volume, and brain age gaps) in participants regularly exceeding the recommended limits of moderate alcohol use versus those who don’t, and to validate self-reported alcohol intake by comparing gamma-GT levels across groups.

**Methods:**

This analysis was based on cross-sectional data from the population-based cohort of the BiDirect Study conducted in Münster (Germany). Individuals aged between 35 and 65 years were randomly selected from the local population register and invited to participate in the assessment that included a 3 Tesla magnetic resonance imaging (MRI) of the brain and a blood collection. Unhealthy alcohol use was defined as the regular consumption of at least three units of alcohol (one unit = 0.2L beer or 0.1L wine or 2cl spirits) per occasion at least twice a week. Regional cortical thickness and subcortical gray matter volumes were extracted from T1-weighted images in participants who underwent MRI. In addition, brain age gaps were estimated using an elastic net algorithm based on the imaging-derived phenotypes. Associations between unhealthy alcohol use, cortical thickness, subcortical gray matter volumes, and brain age gaps were analyzed using multiple regression models adjusted for age, sex, lifetime smoking status, education, and childhood trauma.

**Results:**

Participants engaging in unhealthy alcohol use had significantly higher gamma-GT levels. In addition, unhealthy alcohol use was associated lower regional cortical thickness across all four lobes of the brain. No differences in subcortical gray matter volumes were detected. In addition, we observed a significantly higher brain age gap (+ 1.11 years) in unhealthy alcohol users.

**Conclusions:**

The results of this study indicate that the regular exceedance of the recommended levels of alcohol use is associated with poorer brain health as reflected by lower regional cortical thickness and advanced brain aging. The findings underscore the potentially adverse effects of alcohol on brain health, which are increasingly relevant in view of recent global trends in alcohol use.

**Disclosure of Interest:**

None Declared

